# A multi-information fusion anomaly detection model based on convolutional neural networks and AutoEncoder

**DOI:** 10.1038/s41598-024-66760-0

**Published:** 2024-07-12

**Authors:** Zhongnan Zhao, Hongwei Guo, Yue Wang

**Affiliations:** 1 School of Computer Science and Technology, Harbin University of Science and Technology, Harbin, 150080 China; 2 College of Computer Science and Technology, Harbin Engineering University, Harbin, 150001 China; 3 College of Science, Heilongjiang Institute of Technology, Harbin, 150010 China

**Keywords:** Anomaly detection, Multi-information fusion, CNN, AutoEncoder, Traffic cropping, Electrical and electronic engineering, Computational neuroscience

## Abstract

Network traffic anomaly detection, as an effective analysis method for network security, can identify differentiated traffic information and provide secure operation in complex and changing network environments. To avoid information loss caused when handling traffic data while improving the detection performance of traffic feature information, this paper proposes a multi-information fusion model based on a convolutional neural network and AutoEncoder. The model uses a convolutional neural network to extract features directly from the raw traffic data, and a AutoEncoder to encode the statistical features extracted from the raw traffic data, which are used to supplement the information loss due to cropping. These two features are combined to form a new integrated feature for network traffic, which has the load information from the original traffic data and the global information of the original traffic data obtained from the statistical features, thus providing a complete representation of the information contained in the network traffic and improving the detection performance of the model. The experiments show that the classification accuracy of network traffic anomaly detection using this model outperforms that of classical machine learning methods.

## Introduction

In today’s digital age, cybersecurity has become a crucial aspect of an organization's operations. More and more organizations rely on the internet for their business, and this dependence brings with it the risk of cyberattacks. The methods of cyberattack are constantly evolving, requiring advanced technical measures to provide secure and effective protection^1–3^. Network traffic anomaly detection can help us detect abnormal data traffic in the network, identify unusual patterns or behaviors in network traffic, pinpoint potential security threats, and enable timely countermeasures. Network traffic anomaly detection is a vital area of network security, and designing and selecting the right method can enhance network security.

With the increasing sophistication and complexity of network attacks, traditional methods have become less effective. This has led to the growing use of artificial intelligence techniques, such as machine learning algorithms, in network traffic anomaly detection to detect anomalous information more accurately and efficiently. However, adopting targeted technical means, constructing a corresponding model system, and achieving effective recognition have presented new requirements and challenges for current network anomaly detection. Therefore, the development of continuous technological innovation in the field of anomaly detection has become both a subject of interest and a difficult task.

In recent years, anomaly detection techniques have been pushing the envelope. As machine learning and deep learning-based techniques are increasingly applied and deployed for potential intrusion detection systems, Ahmad et al.^4^ provided a comprehensive review of their advantages and limitations, highlighting the importance of new techniques in this field. Facing the developing trend of unsupervised anomaly detection algorithm research, Roselin et al.^5^ proposed an intelligent optimized deep clustering detection algorithm for extensive network traffic analysis. This algorithm aims to identify and detect unknown malicious traffic without human intervention, representing a significant advancement in the field. Ma et al.^6^ focused on the challenge of designing a detection method for anomalous traffic data based on machine learning. They proposed a novel model called SVM-C, which converts network traffic log information into feature vectors and uses support vector machine classifiers for anomaly determination, thereby forming a machine learning-based approach to characterize traffic analysis. Duan et al.^7^ contributed an extensive and structured survey of fuzzy logic-based methods for detecting network traffic anomalies and distributed denial-of-service attacks. Their work clarifies how fuzzy network anomaly detection methods integrate various techniques, including classifiers and clustering algorithms. Addressing the significant effort required to design and extract feature values in many machine learning and deep learning-based methods, Yoshimura et al.^8^ proposed a classification task based on Perera’s deep one-class classification. By using three different loss functions for training, they simplified the classification of multi-class tagged traffic. Other studies have explored the potential of immune system characteristics in method design. Dutt et al.^9^ proposed a natural immune system model to detect network intrusions, constructing a model that contains both innate and adaptive immune systems. This model forms a screen for the acquisition of initial traffic and available data feature information. Finally, Shi et al.^10^ introduced a novel unsupervised anomaly detection method based on artificial immune networks. Their approach generates rule sets through unsupervised clustering and classification, forming a continuous evolution of self-antibodies and achieving anomaly detection without prior knowledge.

In order to improve and enhance the performance metrics of anomaly detection models, Huo et al.^11^ proposed a comprehensive anomaly detection method that combines improved GRU traffic prediction with enhanced K-means clustering. By linking these techniques in series, their approach aims to boost accuracy and efficiency in anomaly detection while addressing the limitation of K-means clustering in automatically determining cluster numbers. To tackle the issue of low detection accuracy in existing algorithms, Li et al.^12^ leveraged a tensor model for more precise Internet anomaly detection. Their method exploits the multidimensional information hidden in traffic data, exploring the association of data flow information features to enhance detection performance. Addressing misclassification and redundant alarms in traffic classification and prediction, Gao et al.^13^ developed a two-level anomaly detection system. This innovative approach, which integrates deep neural network (DNN) technology with association analysis, seeks to advance the application of DNNs in anomaly detection. Wei et al.^14^ introduced a network anomaly detection method based on deep learning with hierarchical spatio-temporal feature learning. Their approach aims to improve domain feature dependency while simultaneously reducing the high false positive rate often encountered in anomaly detection. Kye et al.^15^ designed a network intrusion detection system that implements hierarchical detection based on self-supervised learning. By combining convolutional neural networks (CNNs) with recurrent neural networks (RNNs), their system automatically learns traffic features, thereby enhancing the accuracy of anomaly detection. Recognizing the challenges of traffic anomaly detection in IoT systems using real-time sensors, Pei et al.^16^ proposed a personalized joint anomaly detection framework. This innovative approach aggregates data while preserving privacy and builds a personalized model through fine-tuning, effectively improving the false alarm rate in these complex environments.

In terms of improving anomaly detection and feature design, Ibrahim et al.^17^ proposed an effective protection mechanism against entropy deception to address the problem of entropy-based anomaly detection methods being susceptible to deception. This mechanism monitors the number of different elements in the feature distribution based on the analysis of changes in different entropy types, making the entropy technique more reliable. Addressing the lack of research on the collective anomaly detection problem in network traffic, Wang et al.^18^ proposed a progressive exploration framework for anomaly detection based on clustering methods to help analysts explore corresponding phenomena. To address the lack of existing models in the dataset and the adaptation of models to the environment, Zhong et al.^19^ proposed a detection framework based on multiple deep learning techniques. This framework updates information and models through the steps of feature extraction, labeled data training, and evaluation, respectively. Duan et al.^7^ analyzed and designed a multi-scale residual classifier (MSRC) based anomaly detection method from the perspective of multi-scale characteristics of network traffic. This method learns data distribution using wavelet change techniques in a sliding window manner. Huang et al.^20^ proposed a multi-channel network traffic anomaly detection method based on decomposition and selection of feature scales, as well as fusion. This approach achieves operational independence of different scales and correlation analysis between multiple scales while reducing computational complexity. Zhang et al.^21^ addressed the imbalance feature problem of attack traffic by framing it as a semi-supervised learning problem. They designed a mutual adversarial network model with two sub-networks using mutual adversarial training to learn normal traffic sample distributions and identify traffic anomaly feature information.

Furthermore, anomaly detection techniques have been widely applied in various fields. In the area of privacy protection in smart grids, Reference^22^ proposed a false data injection attack (FDIA) detection method based on secure federated deep learning and Transformer models, effectively enhancing the security of system data. Reference^23^ focused on FDIA in power systems and utilized a graph convolutional neural network method that integrated time-domain and frequency-domain features to accurately detect and locate attacks. Reference^24^ introduced an anomaly detection method for Internet of Things (IoT) time series, which employed convolutional recurrent AutoEncoders to extract high-level spatial and temporal features, enabling effective detection of anomalies in time series. Reference^25^ proposed an anomaly detection method based on convolutional neural networks, which processed and analyzed concrete structure images to detect defects in the structure. Reference^26^ utilized AutoEncoders and convolutional neural networks for anomaly detection in medical images, identifying abnormal samples by comparing the mean squared error of abnormal data with a baseline threshold. Reference^27^ presented a video anomaly detection method based on deep convolutional AutoEncoders (CAE). In this method, reconstruction errors were used as anomaly scores to identify abnormal events in videos. Reference^28^ designed an autonomous hyperspectral anomaly detection system named Auto-AD, which employed AutoEncoders as core components to learn latent representations of data and detect anomalous pixels in hyperspectral images. These research findings collectively promote the application of anomaly detection techniques in various fields such as smart grids, video surveillance, healthcare, and industrial production. Although these studies have achieved significant results in their respective domains, they mostly focus on single or specific types of data anomalies. Further exploration and improvement are required in handling the integrity of raw data and addressing the complexities of network traffic anomaly detection.

Although numerous existing studies have combined intelligent models such as machine learning and deep learning, leading to improvements in various detection methods and metrics, there is a lack of research on the impact of partial information loss on detection caused by the cropping of raw traffic data. This has hindered the development of effective information models to enhance detection accuracy and performance. To address this gap, this paper proposes a multi-information fusion model based on convolutional neural networks (CNNs) and AutoEncoder (AE). The model utilizes CNNs to extract information from the raw traffic data and AutoEncoder to obtain compressed global information from the statistical features. By merging the high-level information extracted from the raw traffic data and the global information extracted from the statistical features, a new comprehensive feature is formed. This comprehensive feature is then learned through neural networks to obtain a more complete traffic feature representation, ultimately improving the model's detection performance.

The main contributions of this paper are as follows:

1. To address the issue of information loss during the process of cropping raw traffic data, a multi-information fusion model is proposed. By leveraging the fusion characteristics of different models, this approach enables comprehensive acquisition of network traffic information, thereby effectively enhancing the performance of anomaly detection in network traffic.
2. The detection gain effect of multiple information sources is obtained by considering the information classification and recognition abilities of CNN, as well as the feature extraction and data reconstruction characteristics of AutoEncoder. By fully utilizing their respective advantages for local feature learning and statistical feature extraction, richer feature representations are achieved through model fusion, ultimately improving the classification accuracy of the model.
3. Improve the model's robustness and generalization ability, enabling it to perform well across various datasets and scenarios. By leveraging the distinct characteristics and learning abilities of CNN and AutoEncoder, their fusion compensates for the shortcomings and reduces the bias and variance of the model. Consequently, the stability, reliability, and decision-making capability of the anomaly detection model are enhanced.

## Model architecture

**Figure 1:**
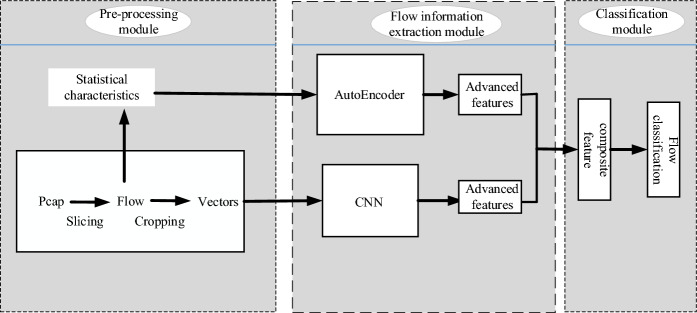
Multi-information fusion model.

A multi-information fusion model based on convolutional neural networks and AutoEncoder (MF-CA) is shown in Fig. 1, and the model structure consists of three main parts: pre-processing module, flow information extraction module, and classification module. In the first part of the pre-processing module, the original pcap traffic will be cropped and sliced. In the second part of traffic information extraction, convolutional neural networks is used to extract high-level features from the original network traffic data, and statistical features are also calculated from the original traffic. AutoEncoder is then used to compress the extracted statistical features. The statistical features do not have to be changed according to the different tasks, they only need to be designed once. The statistical features are mainly used to compensate for the loss of global information due to traffic cropping. Afterwards, we fuse the features extracted by the convolutional neural network with the features obtained by AutoEncoder compression to obtain the combined features. The third part feeds the fused features into the neural network for classification.

## Pre-processing module

**Figure 2:**
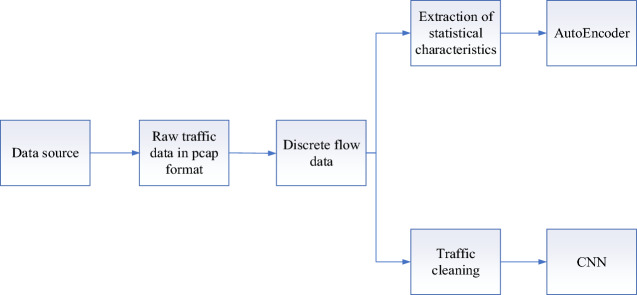
Data pre-processing flowchart.

Because the input to the neural network has a specified format requirement, the raw data needs to be pre-processed first. The initial format of the traffic packets captured in the network is usually pcap format, with the content represented as hexadecimal encoded data, which can be transformed into IPs, ports and other content familiar to security analysts. As shown in Fig. 2, the pre-processing process in this paper is divided into three steps, namely “traffic slicing”, “traffic cleaning” and “statistical feature extraction”.

1. *Traffic slicing* The original pcap traffic file contains a certain number of packets, i.e. $p = \{ p_{1} ,p_{2} , \ldots ,p_{n} \}$,where *n* represents the total number of packets contained in the pcap traffic file. For a single packet ${p}_{i}$, as shown in Eq. (1):
  1 $$ P_{i} = (x_{i} ,t_{i} ,b_{i} ) $$
  where $x_{i}$ represents the five-tuple information, namely source IP, destination IP, source port number, destination port number, and transport layer protocol. $t_{i}$ is the start time of the *i*_th_ traffic packet $p{}_{i}$, and ${b}_{i}$ indicates the size of that traffic pack. Here the SplitCap tool is used to slice the original traffic at the flow level. The network flow $P_{flow}$ represents a flow stitched together from traffic packets with the same quintet of information, as shown in Eq. (2):
  2 $$ p_{flow} = \left\{ {p_{1} = (x_{1} ,t_{1} ,b_{1} ),p_{2} = (x_{2} ,t_{2} ,b_{2} ), \ldots ,p_{n} = (x_{n} ,t_{n} ,b_{n} )} \right\} $$
  where ${x}_{1}={x}_{2}\cdot \cdot \cdot ={x}_{n}$ and ${t}_{1}<{t}_{2}\cdot \cdot \cdot <{t}_{n}$. In the resulting stream, the number of packets in the stream is not the same and varies considerably over a range of timestamps. So we do not use all the packets in the stream. Instead, the first 10 packets in each stream are selected for feature learning.
2. Traffic cleaning: As each network data in the original file contains five network layers of Ethernet layer, network layer, transport layer, and application layer structure. Among them, the MAC source address, MAC destination address and protocol version in Ethernet do not change frequently within the same LAN, and these fields do not contribute to the detection performance of the model in the same network. Therefore, these fields are not used as flow information in this paper. Secondly, the version and different services fields in the network layer also remain almost unchanged in the same network, as we cull these two fields as well. And we find that most of the streams have less than 10 packets, with a small number of streams having more than 10 packets. Since the size of the payload part in each packet is generally different, to make full use of the information in these packets, we selected the first 160 bytes in each packet as the packet feature, using 0 to supplement if it was less than 160 bytes, and cropping if it was more than 160 bytes. In the end, for each stream we extracted a total of 1600 dimensions of raw data.
3. Statistical feature extraction: 26 statistical features are extracted for each stream. For example, the number of packets the stream contains, the duration of the first packet to the last packet in the stream, etc.

## Convolutional neural network module

The Convolutional Neural Network module is responsible for feature extraction from the raw network traffic data. The convolutional neural network requires an input of a specified size, so we make all the traffic the same size by cropping or padding it with zeros. In this paper, the convolutional neural network is used to extract the features of the traffic.

**Figure 3:**
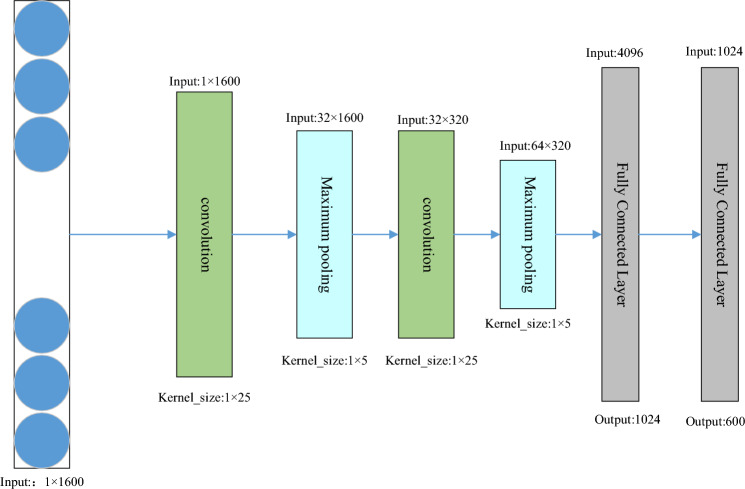
The structure of the CNN.

The convolutional neural network model designed is shown in Fig. 3. The process of feature extraction by convolutional neural network in this paper is as follows.

Firstly, the raw traffic of fixed byte size after cropping is input to the first convolution layer, where the input data size is $\text{V}=\left\{{\text{v}}_{1},{\text{v}}_{2},...,{\text{v}}_{\text{n}}\right\}$, where n is the size of the cropped traffic. 1600 bytes of the network flow is chosen as the input data for the model in this paper. Afterwards, the convolution operation is performed using one-dimensional convolution. The size of the convolution kernel is *1* × *h*. The size of the convolution kernel for the first layer of convolution in this paper is 1 × 25. The convolution kernel operates on a set of traffic bytes and outputs new features. The convolution operation is specified by the following equation:

3 $$ s_{i} = f(w \cdot x_{i:i + h - 1} + b) $$

where *b* is the bias value, *f* is the *Relu* nonlinear activation function, *w* is the parameter that the model updates during training, and the convolution kernel slides over each flow window ${\{x}_{i},{x}_{i+h}\}$ to perform a convolution operation that ultimately produces an output feature mapping.

4 $$ s = [s_{1} ,s_{2} ,...,s_{n - h + 1} ] $$

After the convolution operation, the maximum pooling operation is performed on the feature vector ${s}_{i}$ to reduce the model training parameters, the maximum pooling is to keep the largest feature value in the corresponding feature block as the feature value of the region, the maximum pooling parameter chosen in this paper is 1 × 5 with a step size of 5. The formula for the maximum pooling operation is as follows:

5 $$ \widehat{s} = max(s) $$

The output vector $\widehat{s}$ after pooling is one-fifth of the input vector $s$. It continues to be fed into the next convolutional layer as well as the pooling layer for feature extraction manipulation, and the output feature vector is finally fed into the fully-connected layer to extract the high-level feature $V_{pcap}$. The convolutional kernel of the second convolution and the second pooling layer have the same parameters as the previous convolutional pooling, and after two fully-connected layers, where the output of the first fully-connected layer is 1024 and the output of the second fully-connected layer is 600, are used to and the compressed statistical features are combined into a composite feature. The specific model parameters for the convolutional neural network module are shown in Table 1 below.

**Table 1:** Specific parameters of convolutional neural network.

Network layer	Input	Convolution kernel	Output
Convolution layer-1	1 × 1600	1 × 25	32 × 1600
Maximum pooling	32 × 1600	1 × 5	32 × 320
Convolution layer-2	32 × 320	1 × 25	64 × 320
Maximum pooling	64 × 320	1 × 5	64 × 64
Fully Connected Layer-1	4096	–	1024
Fully Connected Layer-2	1024	–	600

## AutoEncoder module

The AutoEncoder module is responsible for extracting information from statistical features: we extract statistical information from each network flow before performing traffic cropping. These statistics contain some global information that is lost in the network streams due to clipping, such as the “Max pkts length” (Maximum length of traffic packets in the flow), the “Max payload” (maximum value of payload in flow)and the “Num pkt” (the number of packets in the flow). In this paper, 26 statistical features were selected, as shown in Table 2, which are the 26 statistical features extracted in this paper. Among all the statistical features obtained, in order to unify the numerical impact between different magnitudes, this paper adopts the max–min normalisation method for data processing. Equation (6) shows that:

6 $$ x_{i} = \frac{{x - x_{\min } }}{{x_{\max } - x_{\min } }} $$

**Table 2:** 26 statistical features.

No.	Feature name	Description of features
1	Num pkts	Total number of traffic packs streamed
2	Avg ICMP pkt	Average number of ICMP traffic packets in the stream
3	Avg UDP pkt	Average number of UDP traffic packets in the stream
4	Avg DNS pkt	Average number of DNS traffic packets in a stream
5	Avg TCP pkt	Average number of TCP traffic packets in the stream
6	Avg syn flag	Average number of traffic packets with the syn flag bit in the stream
7	Avg urg flag	Average number of traffic packets in the stream with the urg flag bit
8	Avg fin flag	Average number of traffic packets with fin flag bits in the stream
9	Avg psh flag	Average number of traffic packets with the psh flag bit in the stream
10	Avg rst flag	Average number of traffic packets with the rst flag bit in the stream
11	Avg ack flag	Average number of traffic packets with the ack flag bit in the stream
12	Duration windows flow	Holding time for the entire stream
13	Avg delta time	The average size of the incremental time in the flow, the incremental time being the time between two flow packets
14	Min delta time	Minimum value of incremental time in the stream
15	Max delta time	Maximum value of incremental time in the stream
16	StDev delta time	Variance of the incremental time in the stream
17	Avg pkts length	Average size of traffic packets in a stream
18	Min pkts length	The smallest packet size in the stream
19	Max pkts length	Maximum packet size in the stream
20	StDev pkts length	Standard deviation of the flow packet length in the stream
21	Avg small payload pkt	Average number of traffic packets with less than 32 bytes of payload in the stream
22	Avg payload	Average value of payload in stream
23	Min payload	Minimum value of payload in stream
24	Max payload	Max. payload in stream
25	StDev payload	Standard deviation of the payload in the stream
26	Avg DNS and TCP	Share of DNS and TCP in the stream

After the max–min normalisation process, the value domain of the statistical features is [0, 1]. The AutoEncoder compresses these 26 statistical features to obtain some useful information from them. AutoEncoder compresses these 26 statistical features into a 10-dimensional vector space, see Table 2 for details.

**Figure 4:**
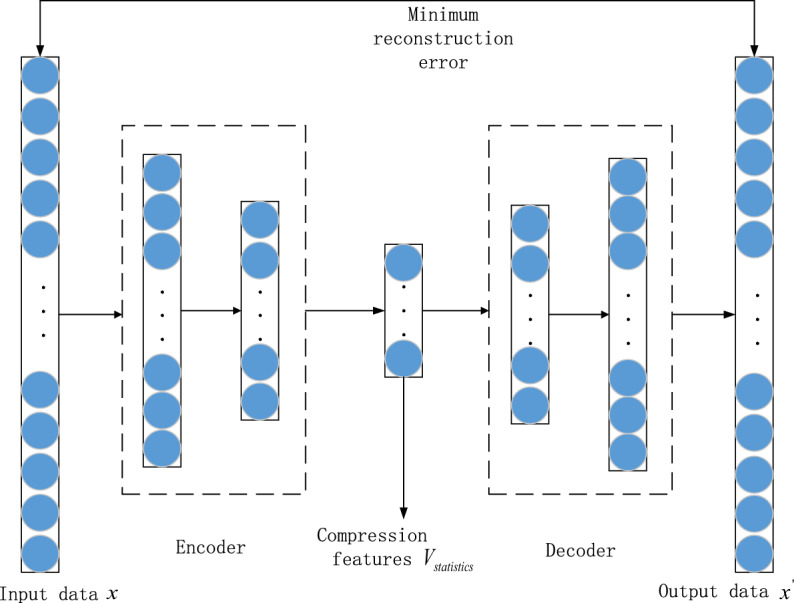
AutoEncoder model diagram.

The extracted statistical features are fed into a AutoEncoder for feature compression, and the AutoEncoder structure used in this paper is shown in Fig. 4.

The input data *S* is feature-coded and compressed by an encoder with the following equation.

7 $$ v = g_{e} (x) = \sigma (W_{1} x + b_{1} ) $$

where $g_{e}$ denotes the nonlinear activation function operation of the input data* x* through the encoder, *W*_1_ denotes the parameter to be learned by the neural network in the encoder, *b*_1_ is the bias value and *σ* denotes the *Relu* activation function.

After encoding by the encoder, a compressed feature *v* is obtained. The compressed feature *v* is fed into the decoder for reconstruction, and the decoder is given the following equation.

8 $$ x{\prime} = g_{d} (v) = \sigma (W_{2} v + b_{2} ) $$

where $g_{d}$ denotes the reconstruction of the compressed feature $v$ through the decoder. *w*_2_ parameters to be learned by the neural network in the decoder, *b*_2_ is the bias value and *σ* is the Relu activation function.

The purpose of the AutoEncoder is to reduce the reconstruction error between the decoder’s output ${x}{\prime}$ to the input data $x$ by continuously optimising the parameters, which in general will be done using Eq. (9).

9 $$ L(x,x{\prime} ) = argmin\frac{1}{m}\sum\limits_{j}^{m} {\left\| {x_{j} - x{\prime}_{j} } \right\|}^{2} $$

Two fully connected neural networks are used for both encoder and decoder respectively, and the specific parameters of the encoder and decoder are listed in Table 3 below.

**Table 3:** Parameters of the AutoEncoder.

Network layer	Network structure	Input	Output
Encoder layer 1	Fully connected layer	26	18
Encoder layer 2	Fully connected layer	18	10
Decoder layer 1	Fully connected layer	10	18
Decoder layer 2	Fully connected layer	18	26

## Classification modules

The advanced features extracted by the convolutional neural network were first spliced with the advanced features compressed by the AutoEncoder, as the original convolutional neural network input was 1600 and the original statistical features were 26. The ratio was approximately 60:1, so we kept the extracted advanced features in this ratio as well and fed the combined features into the classification module for classification. The specific parameters of the neural network for the classification module are shown in Table 4.

**Table 4:** Neural network parameters of the classification module.

Network layer	Input	Output
Fully connected layer	600 + 10	200
Fully connected layer	200	30
Fully connected layer	30	2/8

Where the output of the last layer depends on the classification task, binary classification and octet classification were applied to the data, after which the *softmax* activation function is used for activation. The calculation is shown in Eq. (10):

10 $$ p(c_{i} |n) = \frac{{\exp (s_{i} )}}{{\sum\nolimits_{l = 1}^{r} {\exp (s_{l} )} }} $$

where *p*(*c*_*i*_|*n*) denotes the probability of sample *n* under category ${c}_{i}$, *r* denotes the number of categories, and the sample category is the category with the highest probability.

## Algorithmic process of multi-information fusion model based on CNN and Autodecoder

**Figure 5:**
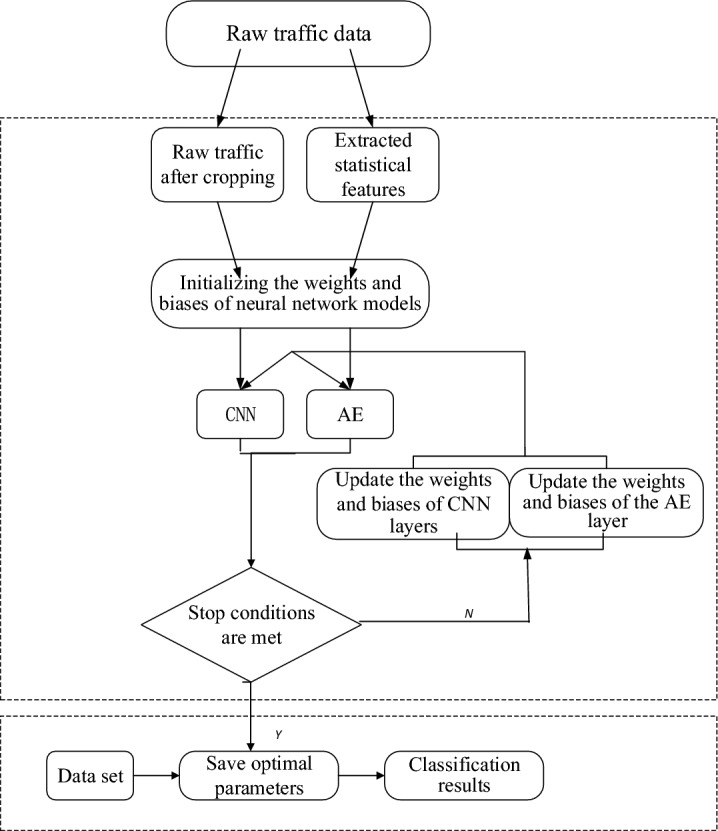
Network traffic anomaly detection algorithm flow chart.

The optimal network parameters saved during model training are used to input the data to be measured into the trained optimal network model, as shown in Fig. 5, which shows the flowchart of the algorithm. The original traffic data is firstly cropped according to the pre-processing method to retain the same size of traffic and extract the statistical features of the original traffic data at the same time, after which the weights of the model are initialized and the cropped traffic is input into the CNN module, and the features are extracted after the calculation of Eqs. (3) to (5), and the statistical features are input into the Autodecoder module and the statistical features are compressed after the calculation of Eqs. (7) to (9) . By continuously training the model and adjusting the model parameters until the end condition is met, and saving the optimal network parameters, the test data is input into the trained model for testing, so as to determine the class to which the test data belongs.

## Experimental results and analysis

### Description of experimental data

This paper utilizes the commonly used CICIDS2017 dataset^29^. As a widely accepted benchmark, CICIDS2017 demonstrates good applicability^30^ and has been extensively employed in research on various network anomaly detection methods^31^. While the dataset providers offer both the original pcap files and a pre-processed version containing 78 statistical features extracted using the CICFlowMeter tool, this paper focuses on processing the original pcap file data. The CICIDS2017 dataset comprises network traffic collected by simulating a real-world attack scenario, spanning five days from Monday, July 3, 2017, to Friday, July 7, 2017. Monday's data consists solely of normal network traffic, while the data from Tuesday to Friday includes instances of DDOS, brute force FTP, and botnet attacks. The network traffic is accurately labeled based on five-tuple information for each flow.

Normal traffic as well as 10 types of attack traffic were extracted from the original dataset as the test and training sets for the model under study. The data pre-processing process is described in detail. The final labels of the data we extracted and the corresponding quantities are shown in Table 5. From Table 5 it can be seen that the data samples labelled Normal and Port Scan far exceed the number of other samples. To prevent imbalance in classification accuracy due to too many samples of these two types of labels, these two types of samples are randomly undersampled during the octet classification experiments, with 10,000 sample data reserved for each type. In contrast, no undersampling was performed for the binary classification.

**Table 5:** CICIDS2017 label classification and quantity distribution.

Types of streams	Original quantity
Normal	339,621
Dos	
DoS GoldenEye	7458
DoS Hulk	14,108
DoS Slowloris	4216
DoS Slowhttp	3869
Web attack	
Brute Force	1353
SQL Injection	12
XSS	631
SSH Patator	2511
FTP Patator	3907
BotNet	1441
Port Scan	158,673
DDos	16,050

**Figure 6:**
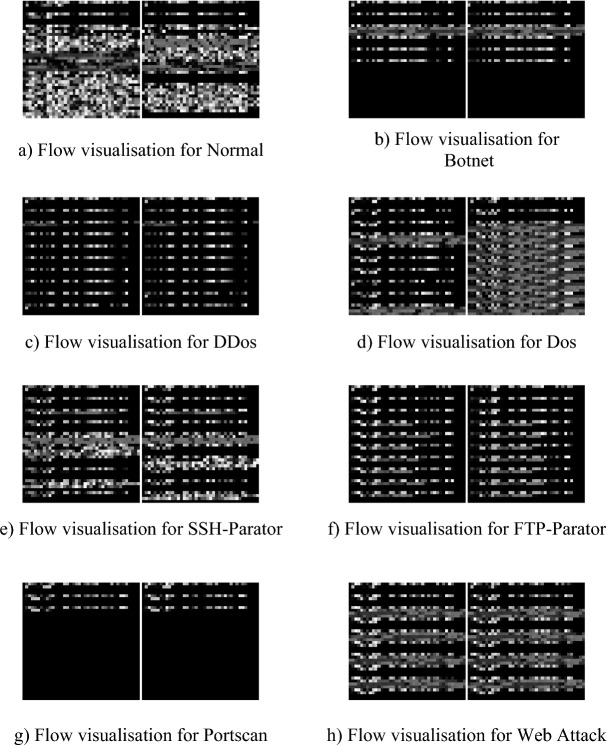
Visualization of 8 types of samples in CICIDS2017 dataset.

The original traffic was pre-processed and turned into 1600-dimensional data. For visualisation, the feature length of 1600 dimensions was transformed into a 40 × 40 matrix, and Fig. 6 shows the grey-scale map of the eight types of samples. From Fig. 6 it can be seen that there are clear differences between the different samples, for example Normal and Botnet labels, where it is easy to see that the texture differs between the two samples, but there are also samples that are more similar, for example the two samples DDos and FTP-Parator labels, where there are only subtle differences between them.In contrast, samples of the same species all have a similar distribution between them, for example the Portscan and Web Attack labels. In summary, there is a large difference between samples from different labels, while samples from the same label have relatively similar textures.

### Analysis of experimental results

The performance evaluation metrics for the models use accuracy, precision, recall and F1 score. Convolutional neural network models using only raw traffic data, AlexNet, ResNet, and some classical machine learning models were selected for binary Classification and octet classification experiments. In order to ensure that all category samples are equally distributed between the test and training sets, we select 70% of the total data from each label as the training set and 30% as the test set. The coding table for the classification labels is shown in Table 6.

**Table 6:** CICIDS2017 classification label coding table.

Label	Code
Normal	0
Dos	1
SSH	2
FTP	3
BotNet	4
Port Scan	5
DDos	6
Web Attack	7

#### Determination of model parameters

**Figure 7:**
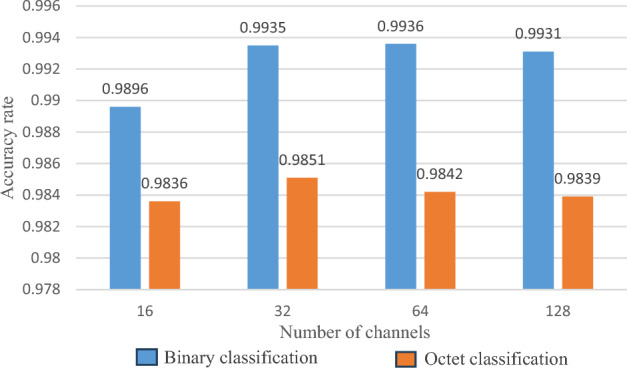
Classification results of different channel numbers.

Some parameters of the convolutional neural network were experimentally tuned and the performance of the model was largely determined by the parameters of the neural network. Firstly, the number of convolutional channels was experimentally analysed to find the optimal number of convolutional channels. The number of convolutional channels in the first layer was set to 16, 32, 64 and 128, respectively, to compare the effect of the number of channels on the classification accuracy of the model, while controlling other parameters constant. The experimental results are shown in Fig. 7.

From Fig. 7, it can be seen that in binary classification, the classification accuracy gradually increases when the number of convolutional channels is 16, 32 and 64, and starts to decrease when the number of channels is 128. And at the number of channels of 64, the accuracy only increases by 0.01%, but it takes more training time. At octet classification. The number of channels already starts to decrease at 64. Therefore, combining performance and cost considerations, the model proposed is finally chosen to have a first layer of 32 channels.

Secondly, in order to compare the effect of different network layers in CNN on the performance of the model, we selected the number of layers of convolutional layers from one to four and conducted accuracy comparisons, keeping other parameters constant, and the experimental results are shown in Table 7.

**Table 7:** Classification results of different convolutional layers.

The number of convolutional layers	Binary classification	Octet classification
one layer	0.9810	0.9801
two layers	0.9935	0.9851
three layers	0.9937	0.9847
four layers	0.9931	0.9839

As can be seen from Table 7, the model with one layer of convolution performs the worst. At binary classification, the performance of the three-layer convolution is slightly better than that of the two-layer convolution, and the accuracy of the model starts to drop when the number of layers reaches four; at multiple classification, the accuracy of the model starts to drop already at three layers of convolution. Although the performance of the three-layer model is somewhat better when it comes to binary classification, deeper layers mean more parameters and higher learning costs. Combined performance and cost considerations, the two-layer convolutional neural network model was finally chosen as our layer in this paper.

Finally, to explore the effect of 1D and 2D convolution on model performance, we also tested the difference in classification accuracy of the model at octet classification for different layers of 1D and 2D convolution.

From Table 8, it can be found that the classification accuracy of the model with two-dimensional convolution at any number of layers is lower than that of the one-dimensional convolutional neural network model.

**Table 8:** Comparison of octet classification performance between different dimensional convolutions.

The number of convolutional layers	One-dimensional convolution	Two-dimensional convolution
One layer	0.9801	0.9742
Two layers	0.9851	0.9811
Three layers	0.9847	0.9805
Four layers	0.9839	0.9830

#### Binary classification experiments

The classification results of this paper on the CICIDS2017 dataset using classical machine learning models such as CNN and Random Forest are presented in Table 9. In this paper, This CNN1D model, which is the CNN model applied in this paper, employs one-dimensional convolution to directly extract features from the raw traffic without combining statistical features compressed by the AutoEncoder. As can be seen from Table 9, the deep learning models generally outperformed the traditional machine learning models when binary classification was performed on the CICIDS2017 dataset, all achieving an accuracy of over 99%, and the model proposed in this paper performed the best of all models, reaching the highest values in all four metrics. The SVM had the worst performance among the models.

**Table 9:** The results of the binary classification experiment of each model on CICIDS2017.

Model	Accuracy	Precision	Recall	F1-Score
CNN1D	0.9901	0.9902	0.9901	0.9901
DT	0.9653	0.9666	0.9653	0.9655
RF	0.9681	0.9692	0.9681	0.9682
SVM	0.9535	0.9545	0.9535	0.9536
KNN	0.9663	0.9664	0.9663	0.9663
AlexNet	0.9908	0.9909	0.9908	0.9908
ResNet	0.9910	0.9909	0.9910	0.9910
MF-CA	0.9935	0.9935	0.9935	0.9935

#### Multi-classification experiments

To further validate the performance of the model, an experimental study of octet classifications was conducted on the CICIDS2017 dataset. As shown in Table 10, the experimental results of octet classification are presented. From Table 10, it can be found that the performance of the three deep learning models, AlexNet, ResNet and CNN1D, still outperformed the traditional machine learning models in octet classification, while among the traditional machine learning models, KNN had the worst performance. The model proposed achieved 98.51% accuracy, 98.31% precision and 98.31% F1 score, which were the highest among all models with the highest values.

**Table 10:** Comparison of the octet classification experiment results of each model on the CICIDS2017 dataset.

Model	Accuracy	Precision	Recall	F1 Score
CNN1D	0.9786	0.9769	0.9786	0.9764
DT	0.9653	0.9638	0.9653	0.9630
RF	0.9682	0.9667	0.9682	0.9656
SVM	0.9435	0.9439	0.9435	0.9411
KNN	0.9357	0.9245	0.9357	0.9308
AlexNet	0.9790	0.9771	0.9790	0.9770
ResNet	0.9801	0.9783	0.9801	0.9781
MF-CA	0.9851	0.9831	0.9851	0.9831

**Figure 8:**
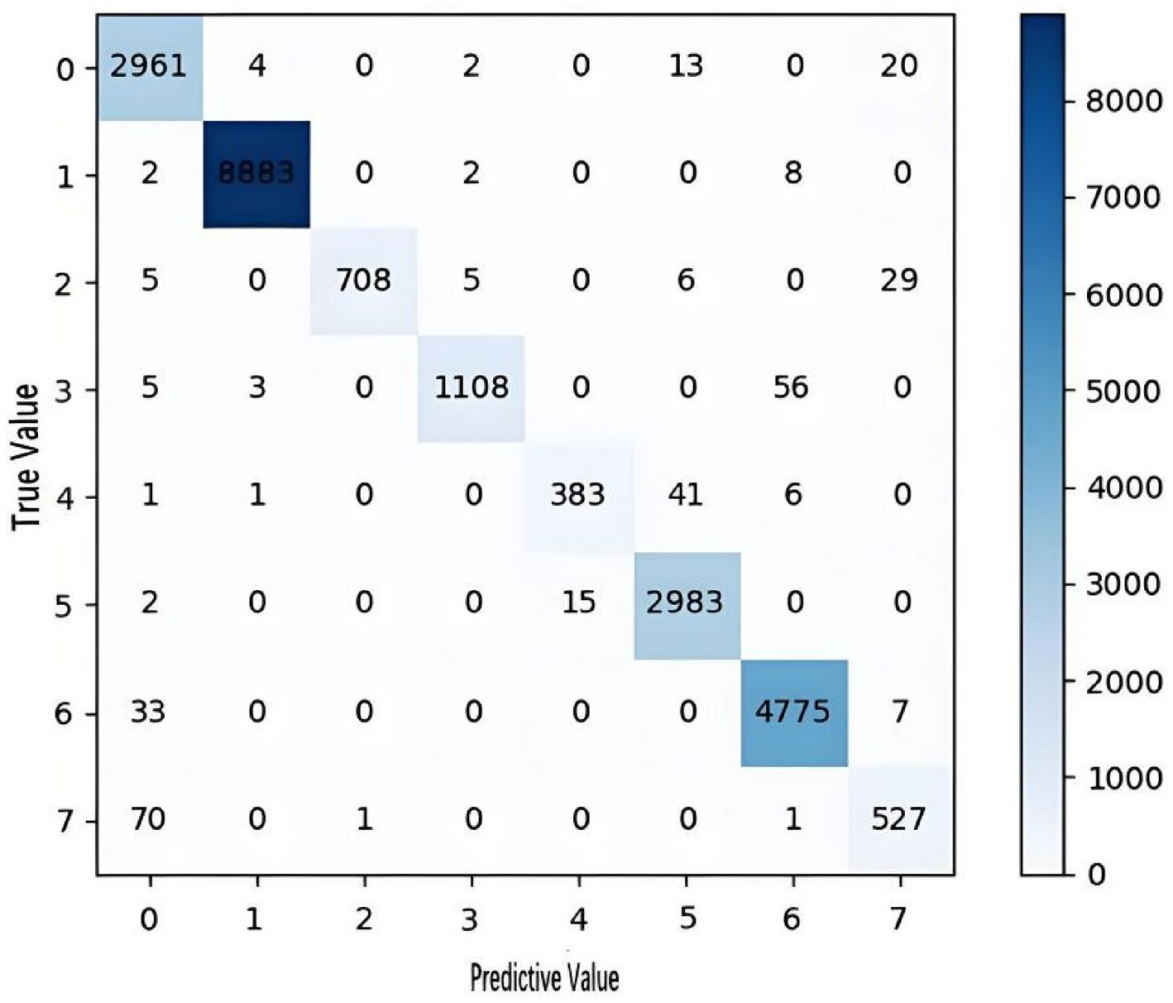
Confusion matrix of the CICIDS2017 dataset.

To clearly show how well the model predicted the samples for each class, we plotted the confusion matrix shown in Fig. 8. As can be seen in Fig. 8, we classified most of the samples correctly. The majority of the incorrectly predicted samples of label 4 were predicted to sample 5. As can be seen from the grey scale plot, there are indeed many similarities between the two labelled samples, which makes it more difficult to classify the model and may lead to misclassification of the model.

**Figure 9:**
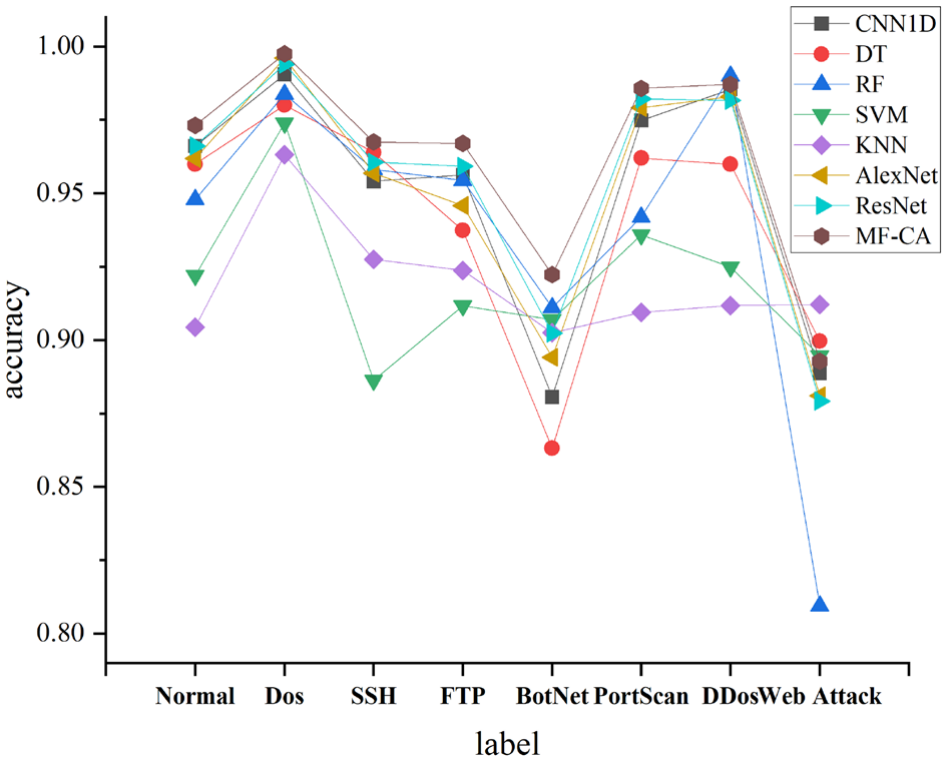
Comparison of F1 scores in each category of each model.

**Figure 10:**
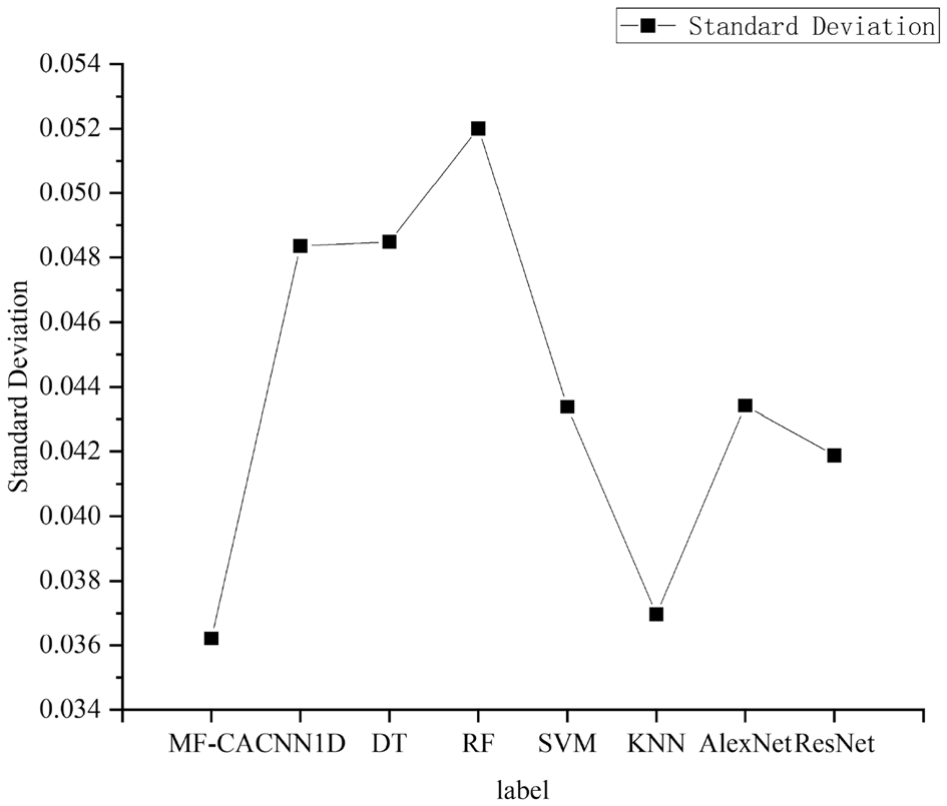
Standard deviation.

**Figure 11:**
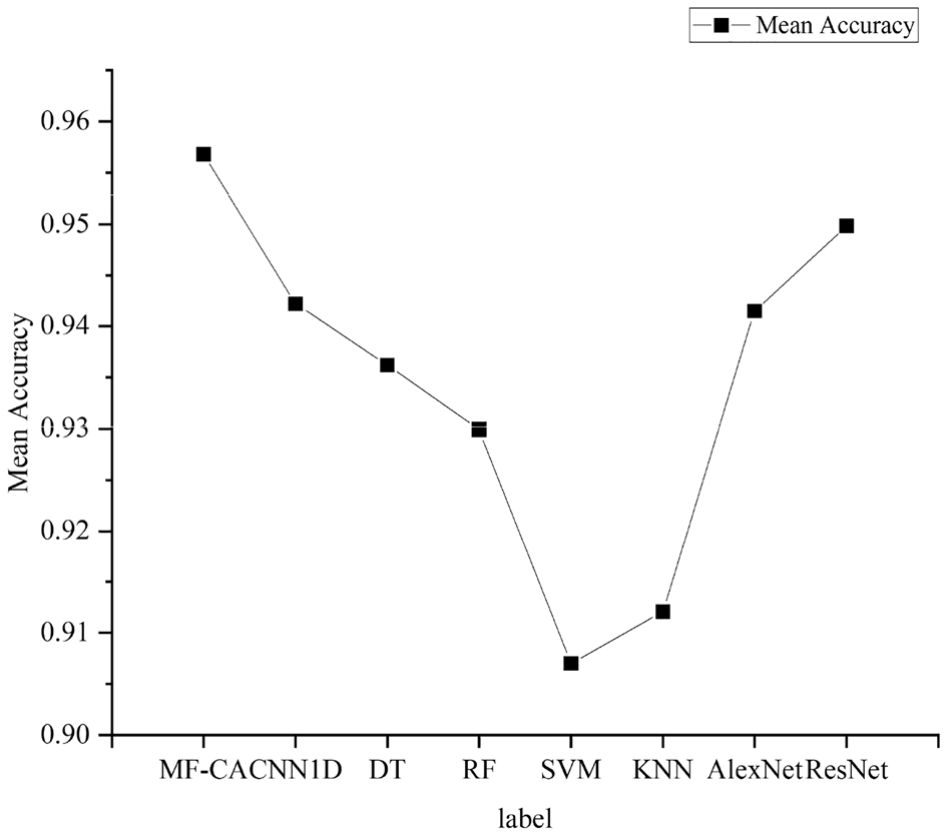
Mean accuracy.

To compare the classification performance of the models on different types, we plotted the F1 scores of the models on each category. As shown in Fig. 9, it can be seen from the graph that the model proposed achieves the best F1 scores on most labels, and overall performance is the best. To facilitate the analysis of the test performance of the proposed algorithm in this paper, additional statistical analyses were conducted. The standard deviation and mean accuracy are presented in Figs. 10 and 11, respectively. It is worth noting that the proposed algorithm in this paper achieves the smallest standard deviation and highest average accuracy, indicating its superior robustness and generalization ability.

## Analysis and summary

To facilitate a deeper understanding of the model's architecture, functionality, implementation details, and relevant characteristics, the following section will provide further analysis and discussion.

Firstly, regarding the model’s structural design. In this study, the model's input source data from the CICIDS2017 dataset. The raw data is stored in pcap format, recording a series of raw byte streams of network packets. By parsing these pcap files, relevant information about network flows was extracted and based on this, the traffic data was segmented into individual flows for model feature processing. Feature engineering is a critical step to ensure model performance. Initially, the raw pcap data was cleaned by removing unnecessary addresses and fields. Then, the data for each flow was cropped to ensure all input data had a uniform dimension. To further extract useful global information, an AutoEncoder was used to compress the statistical features. Finally, high-level features extracted by the convolutional neural network are combined with AutoEncoder-compressed statistical features to form comprehensive features, capturing more extensive traffic information. During the comprehensive evaluation of the model, particular attention was paid to its accuracy, generalization ability, and robustness. To measure these performance metrics, accuracy, precision, recall, and F1 score were used as the primary evaluation standards. These metrics not only reflect the model's performance on a specific dataset but also provide a reference for its potential performance in practical applications.

The experimental results demonstrate that the model achieved high accuracy in various classification tasks. These results not only demonstrate the model's good accuracy on the current dataset but also indicate that the model has excellent generalization ability and can adapt to different network traffic patterns. Meanwhile, the model's generalization ability was also validated in tests involving different types of network attack scenarios. The model performed well not only on known attack types but also maintained high detection accuracy when faced with unknown attack types, further proving the model's good generalization performance. Additionally, through the analysis of the confusion matrix, the model's performance in various categories was further observed, which helps in understanding the model's decision-making process. In terms of evaluating the model's robustness, particular attention was given to its performance when faced with noise and outliers in the dataset. Testing with complex environmental data revealed that the model maintained high performance even in the presence of incomplete data or noise, demonstrating its robustness. The stability of key performance metrics such as accuracy, precision, recall, and F1 score under different testing conditions further highlights the model's robustness in the face of different attack patterns and data perturbations. Combining these evaluation results provides a comprehensive understanding of the model's performance. The model not only performs well on the current dataset but also has the potential for application in a broader network environment.

Feedback and Decision-making. The model's effectiveness is reflected not only in experimental results but also in its positive impact on real network environments. If the model undergoes further validation and is accepted, plans involve integrating its feedback loop into actual network environments to assist network administrators and security analysts in making more informed decisions. The integration process will include the following key steps: First, the model will be deployed in network traffic monitoring systems to analyze incoming data packets in real-time and identify potential anomalous behaviors. Second, once an anomaly is detected, the model will provide detailed alert information, including the type of anomaly, its severity, and possible impacts. A good feedback mechanism can not only help users identify and respond to current security threats but also effectively enhance the overall security of the network environment.

Secondly, regarding parameter optimization. To determine the optimal hyperparameters for the Convolutional Neural Network (CNN) and Autoencoder models, this paper employed Bayesian optimization. Bayesian optimization is an efficient global optimization strategy that guides the search process by constructing a probabilistic model of the hyperparameters, thereby finding the optimal solution with fewer evaluations. This paper defined a prior distribution to express the initial uncertainty about the hyperparameters and updated the posterior distribution based on the results of each model evaluation. Through this method, it was possible to gradually narrow down the search range and ultimately determine the hyperparameter combination that maximizes model performance. The specific implementation included the following steps: First, learning rate, number of network layers, and number of hidden units were selected as the target hyperparameters for optimization. Then, a search space was defined for each hyperparameter, and Gaussian processes were used to model the relationship between hyperparameters and model performance. In each iteration, the most promising hyperparameter combination was selected for evaluation based on the posterior distribution, and the model was updated. By repeating this process, the hyperparameter settings that performed best on the validation set were ultimately found. The reason for choosing Bayesian optimization lies in its efficiency and effectiveness in handling high-cost function evaluations, which is particularly important for hyperparameter tuning of deep learning models. Through Bayesian optimization, not only was the efficiency of the model tuning process improved, but the model’s performance in anomaly detection tasks was also ensured.

Furthermore, to prevent overfitting, this paper adopts several measures to reduce the risk of model overfitting and ensure model performance. First, data preprocessing and normalization steps are implemented to clean the data and reduce noise interference. Then, L2 regularization is introduced to effectively control model complexity, limiting the model's tendency to overfit. Additionally, early stopping is applied during training, terminating the process when validation set performance improvements stagnate, preventing the model from overfitting the training data. Simultaneously, hyperparameter tuning is performed using Bayesian optimization methods to optimize parameter combinations, avoiding complex model data noise, and improving model generalization performance while reducing overfitting risk. Moreover, the model's performance metrics on both the training and validation sets are continuously monitored to ensure balanced model behavior. By combining these measures, the risk of overfitting is effectively reduced, ensuring that the model demonstrates better detection performance when dealing with complex network traffic.

Finally, regarding the applicability and foresight of the model. In the face of the dynamic nature of the cybersecurity environment, it is crucial for a model to possess foresight and adaptability in handling evolving network threats. This paper has specifically considered these aspects in the model design. The following are the key features of the model that enable it to adapt to new threats. Multi-Feature Fusion Strategy: The model employs a multi-feature fusion strategy, extracting both local features from raw traffic data and global information from statistical features. This fusion provides a comprehensive understanding and deep insight into network traffic, significantly enhancing the ability to identify emerging and unknown anomalous patterns. The integration of an AutoEncoder enhances the model's reconstruction capability, allowing it to detect new anomaly patterns by capturing the intrinsic structure of the data. Scalability of the Model Architecture: The design of the model architecture emphasizes scalability, allowing the integration of new detection technologies and algorithms. Future research particularly considers the model's capability for incremental training. This design enables the model to be updated in a modular fashion, ensuring continuous optimization of model performance through environmental readaptation. This allows the model to quickly adapt to new network threats, forming a long-term mechanism for security response.

## Conclusion

In order to avoid the loss of traffic data information in the process of network anomaly detection and to improve the detection performance of traffic feature information, a multi-information fusion model based on CNN and AE is proposed. The method can combine the load information contained in the raw traffic data and the global information contained in the statistical features, and fuse them to obtain more complete and comprehensive information, thus allowing better feature representation and enhancing the ability to identify network traffic anomalies. The performance of our model was tested on the CICIDS2017 dataset. The results show that the method proposed in this paper achieves 99.35% accuracy in binary classification and 98.51% accuracy in octet classification, which is higher than deep learning models using only raw traffic data and classical machine learning models.

## Data Availability

CICIDS2017: https://www.unb.ca/cic/datasets/ids-2017.html.
